# Effective approaches to discover new microbial metabolites in a large strain library

**DOI:** 10.1093/jimb/kuab017

**Published:** 2021-02-18

**Authors:** Mitja M Zdouc, Marianna Iorio, Kristiina Vind, Matteo Simone, Stefania Serina, Cristina Brunati, Paolo Monciardini, Arianna Tocchetti, Guadalupe S Zarazúa, Max Crüsemann, Sonia I Maffioli, Margherita Sosio, Stefano Donadio

**Affiliations:** NAICONS Srl, 20139 Milan, Italy; Swammerdam Institute for Life Sciences, University of Amsterdam, 1098 XH Amsterdam, The Netherlands; NAICONS Srl, 20139 Milan, Italy; NAICONS Srl, 20139 Milan, Italy; Host-Microbe Interactomics Group, Wageningen University, 6708 WD Wageningen, The Netherlands; NAICONS Srl, 20139 Milan, Italy; NAICONS Srl, 20139 Milan, Italy; NAICONS Srl, 20139 Milan, Italy; NAICONS Srl, 20139 Milan, Italy; NAICONS Srl, 20139 Milan, Italy; Institut für Pharmazeutische Biologie, Rheinische Friedrich-Wilhelms-Universität, 53115 Bonn, Germany; Institut für Pharmazeutische Biologie, Rheinische Friedrich-Wilhelms-Universität, 53115 Bonn, Germany; NAICONS Srl, 20139 Milan, Italy; NAICONS Srl, 20139 Milan, Italy; NAICONS Srl, 20139 Milan, Italy

**Keywords:** Actinomycetes, Antibiotics, New RiPP family, *Planomonospora*, Pseudouridimycin, *Streptomyces*

## Abstract

Natural products have provided many molecules to treat and prevent illnesses in humans, animals and plants. While only a small fraction of the existing microbial diversity has been explored for bioactive metabolites, tens of thousands of molecules have been reported in the literature over the past 80 years. Thus, the main challenge in microbial metabolite screening is to avoid the re-discovery of known metabolites in a cost-effective manner. In this perspective, we report and discuss different approaches used in our laboratory over the past few years, ranging from bioactivity-based screening to looking for metabolic rarity in different datasets to deeply investigating a single *Streptomyces* strain. Our results show that it is possible to find novel chemistry through a limited screening effort, provided that appropriate selection criteria are in place.

## Introduction

Natural products have made a significant impact on our well-being by providing many molecules to treat and prevent illnesses in humans, animals and plants (Newmann & Cragg, 2020). A general consensus exists that, having sampled just a small fraction of the existing microbial diversity with a limited number of bioactivity-based screens, there is plenty of unexplored chemical space from microbial sources (Genilloud, 2017; Wright, 2017). Nonetheless, the main challenge in microbial metabolite screening is avoiding the re-discovery of known molecules, which rank in the tens of thousands (van Santen et al., 2021). To this end, different approaches have been implemented that essentially rely on the combination of two elements: an appropriate source of microbial diversity on the one hand, such as existing strain collections, unexplored microbes or environmental DNA; and a way to screen these sources, whether by genome mining, bioactivity screening or chemical profiling (Monciardini et al., 2014; Steele et al., 2019; van Bergeijk et al., 2020). While each of the above approaches has advantages, we still do not know the most cost-effective way of proceeding for a desired objective.

In this perspective, we describe our recent experience in looking for novel microbial metabolites by evaluating a small portion of our strain collection of about 45 000 actinomycetes. Four different examples are reported, all highlighting the need for effective databases and data management.

## The Strain, Extract, and Fingerprint Libraries

**Fig. 1:**
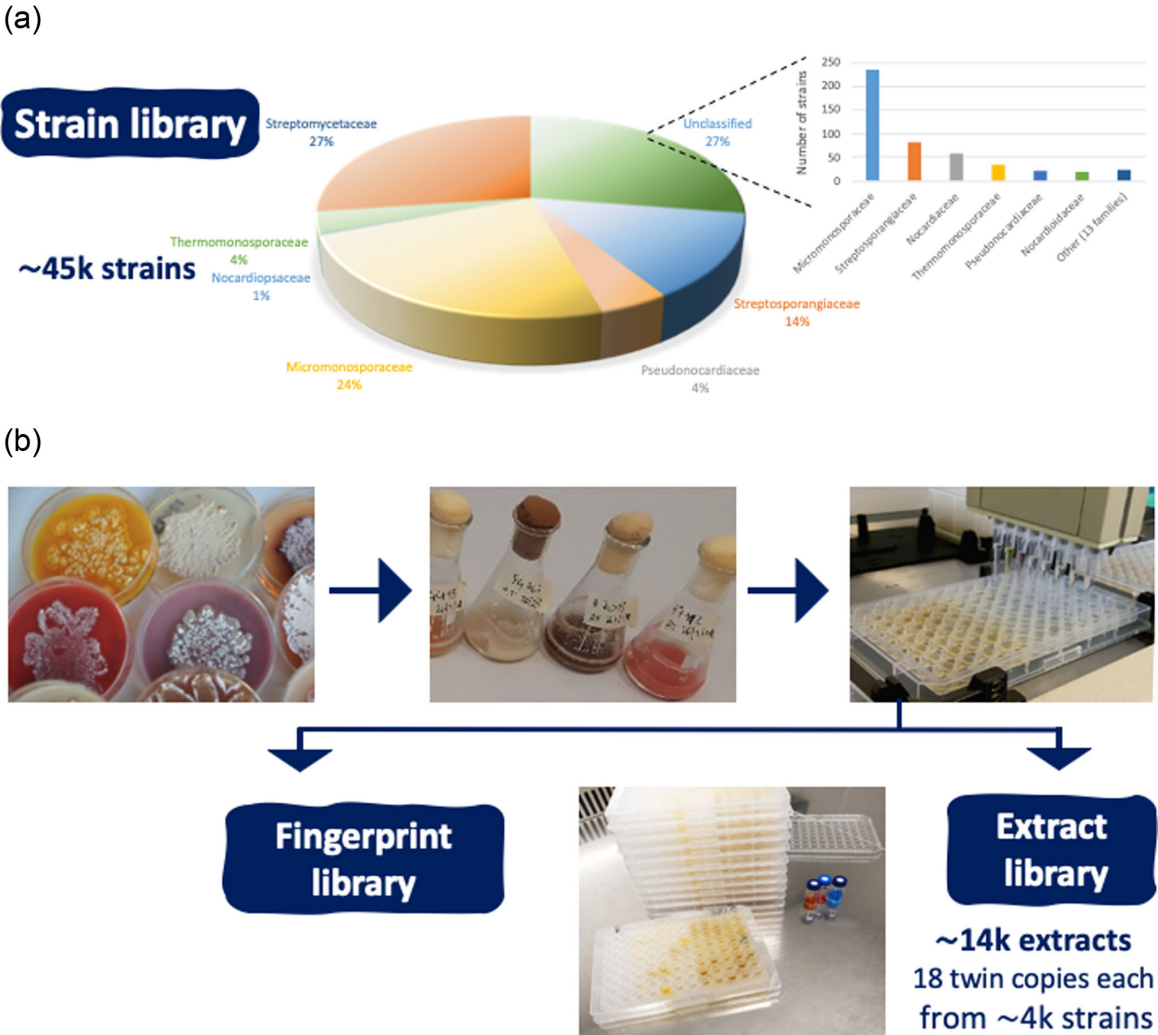
The NAICONS strain (a) and extract libraries (b) as of September 2020. (a) The top portion shows the distribution of strains at family level as previously reported (Monciardini et al., 2014). The enlargement shows the distribution into families of an “unclassified” portion of the strain library, as detailed in the text. (b) Schematic flow for the preparation of the extract library and the generation of the LC–MS fingerprints.

The NAICONS library consists of about 45 000 strains (Fig. 1a), mostly filamentous actinomycetes isolated over a 10-year period by NAICONS predecessor company and classified mostly on the basis of morphology (Monciardini et al., 2014). Only a small fraction of the library has been evaluated for chemical novelty by focusing on strains belonging to the unusual genera *Actinoallomurus* (Iorio et al., 2018) and *Planomonospora* (see below). We undertook an effort by targeting the most abundant portions of the library, namely: strains belonging to the *Streptomycetaceae*; and those listed as “unclassified” (Fig. 1a). *Streptomyces* spp. are known for their ability to produce a great number of specialized metabolites, grow relatively rapidly and cultures from production media can be harvested after a 3-day cultivation, speeding up the process for extract preparation. Conversely, the “unclassified” portion of the strain library was expected to be heterogeneous. Indeed, 16S-based genus assignment of just 4% of the “unclassified” portion of the library indicated that the analyzed strains belong to 19 different families, as shown in Fig. 1a.

Strains were systematically retrieved, streaked on solid medium and cultivated in one or two media. After collecting the biomass by centrifugation, two extracts per culture were prepared following previously reported procedures (Donadio et al., 2009): the mycelium was extracted with ethanol, while the cleared broth was adsorbed on a resin and released by elution with methanol. Parallel processing of 40 cultures yielded 80 extracts, which were dispensed into the wells of 18 twin 96-well microtiter plates, dried and stored for further use. Repetition of these procedures resulted in the generation of 180 sets of microtiter plates, corresponding to about 14 000 different extracts derived from approximately four thousand strains (Fig. 1b). Each extract was analyzed by liquid chromatography-mass spectrometry (LC–MS), providing a dataset of molecular fingerprints (Fig. 1b).

The extracts and the molecular fingerprints represent a valuable asset to search for novel chemistry, as they provide a reasonably sized dataset, as detailed in the examples reported below.

## Bioassay-Based Screening

Bioassay-based screening has been traditionally employed in microbial product screening, since it can quickly pinpoint desired activities for follow-up investigations. Nonetheless, it represents the most challenging approach, especially when looking for antimicrobial activities. Indeed, it can be estimated that, over several decades and across many academic and industrial institutions, tens of millions of strains have been screened.

Screening about 11 000 extracts for growth inhibition of a clinical isolate each of *Klebsiella pneumoniae* and *Acinetobacter baumannii* led to 1.1, 1.9, and 1.3% samples inhibiting *K. pneumoniae, A. baumannii* or both, respectively. Active extracts were then HPLC-fractionated, the active fractions were identified by bioassay and the MS, MS/MS, and the UV–Vis spectrum associated with the active fractions were compared to internal and external databases, in a process known as “dereplication.” Results are most advanced for the *K. pneumoniae*-inhibiting samples, as described below.

**Fig. 2:**
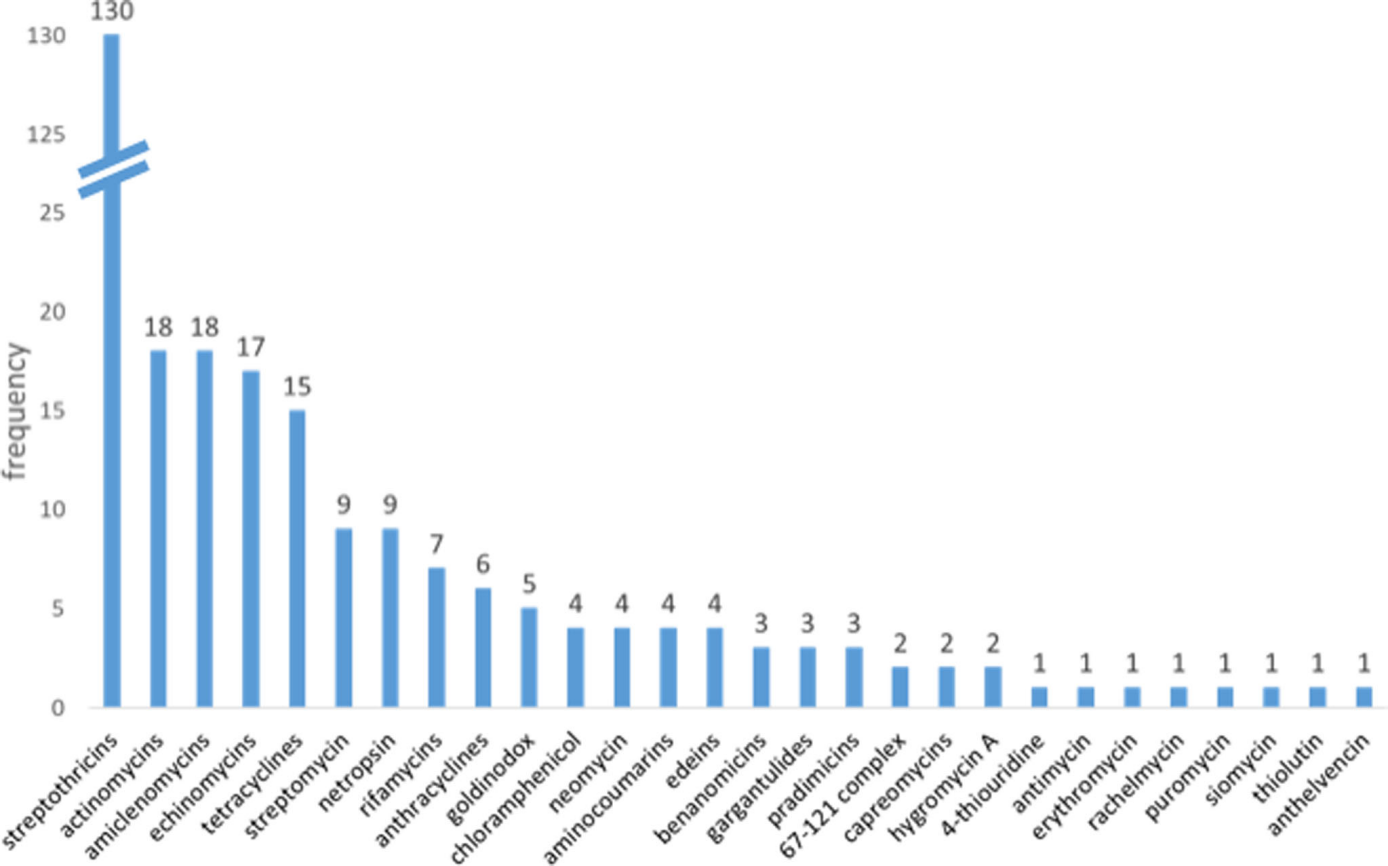
Frequency of molecules dereplicated in about 300 extracts inhibiting growth of either *Klebsiella pneumoniae* and/or *Acinetobacter baumannii*. Each bar may represent different metabolite belonging to the same molecular family.

So far, we have dereplicated most of the extracts inhibiting growth of *K. pneumoniae* only and about half of the extracts inhibiting growth of either *A. baumannii* only or of both, leading to 23 molecular families (Fig. 2). The most frequently encountered was streptothricin and related *N*-glycosylated metabolites containing an ε-linked peptidyl chain of β-lysine units. Originally described in 1942 (Waksman & Woodruff, 1942), this family accounted for over one third of the hits. Other frequent metabolite families were: the amiclenomycins, di- or tripeptides carrying a 4-amino-2,5-cyclohexadienyl moiety that act by blocking biotin biosynthesis (Kern et al., 1985); edeins, broad-spectrum peptide antibiotics carrying a (guanidinylated)-spermidine unit at the C-terminus and first isolated from *Bacillus brevis* (Hettinger & Craig, 1970); netropsin and related pyrrolamides, DNA-binders isolated from different *Streptomyces* spp. (Kopka et al., 1985); and the clinically used antibiotics streptomycin and oxytetracycline. All together five metabolite families accounted for almost 80% of the hits. This is a striking example of the uneven distribution of metabolites in screened libraries, as previously reported (Baltz, 2019), and of the loose correlation between frequency of events and history of antibiotic discovery. It should be noted that over 90% of hits derived from *Streptomyces* spp., while the remaining were distributed among several different genera. While this uneven distribution might be related to the presence of nearly identical strains in the screened set, this is a quite common occurrence in large strain libraries put together before the genomic era.

When looking at genus level, hit rates were higher for *Streptomyces* spp., with 4.0% of the strains tested producing one or more active extracts. In comparison, the hit rates among all other genera were a mere 0.9%. However, when filtering out the two most frequent hits, streptothricins and amiclenomycins, the hit rate of *Streptomyces* isolates was reduced to 1.9%, only twice as high as all other genera. It should be noted that nuisance compounds can be filtered out by appropriate tests. For example, we observed that a *K. pneumoniae* strain carrying the nurseothricin resistance gene *nat* (Krügel et al., 1988) on a multicopy plasmid becomes fully resistant to streptothricin and related compounds.

Overall, this analysis indicates that, at least for screening of streptomycetes and other relatively common actinomycetes for antibacterial activities, the expectation is that new compounds will represent a rare event, and that most hits will be generated by relatively frequent nuisance compounds. Consequently, when looking for compounds active against Gram-negative bacteria, a hit rate around 1–2%—after excluding the most frequent classes, as mentioned above—implies a large screening effort to have a reasonable number of rarely occurring metabolites among which to find novel and potentially useful compounds.

## Rare Events in Common Strains

Bioactivity-based screens, such as looking for antibacterial activity, impose one technical and one historical bias as selection criteria: technically, only compounds active against the target strain(s) and present at a concentration high enough to inhibit growth will be detected; historically, the screening for bacterial growth inhibition by actinomycete cultures has been performed extensively, thus reducing the chances of finding new chemistry in a relatively small number of strains.

We were also interested in assessing the chemical novelty existing in our extract collection. To this end, we are exploring the possibility of enhancing signals by cultivating strains under a variety of conditions, including the presence of elicitors or stress substances. In a pilot experiment, we examined 21 diverse *Streptomyces* isolates from our collection under 20 different conditions. While this approach did not significantly alter the frequency of antibacterial activities, consistent with larger studies of this sort (Okada & Seyedsayamdost, 2017), the resulting collection of extracts and associated LC–MS fingerprint provided an excellent opportunity for investigating whether any of those 21 strains reliably produced metabolites not observed in the 14 000 NAICONS extracts.

**Fig. 3:**
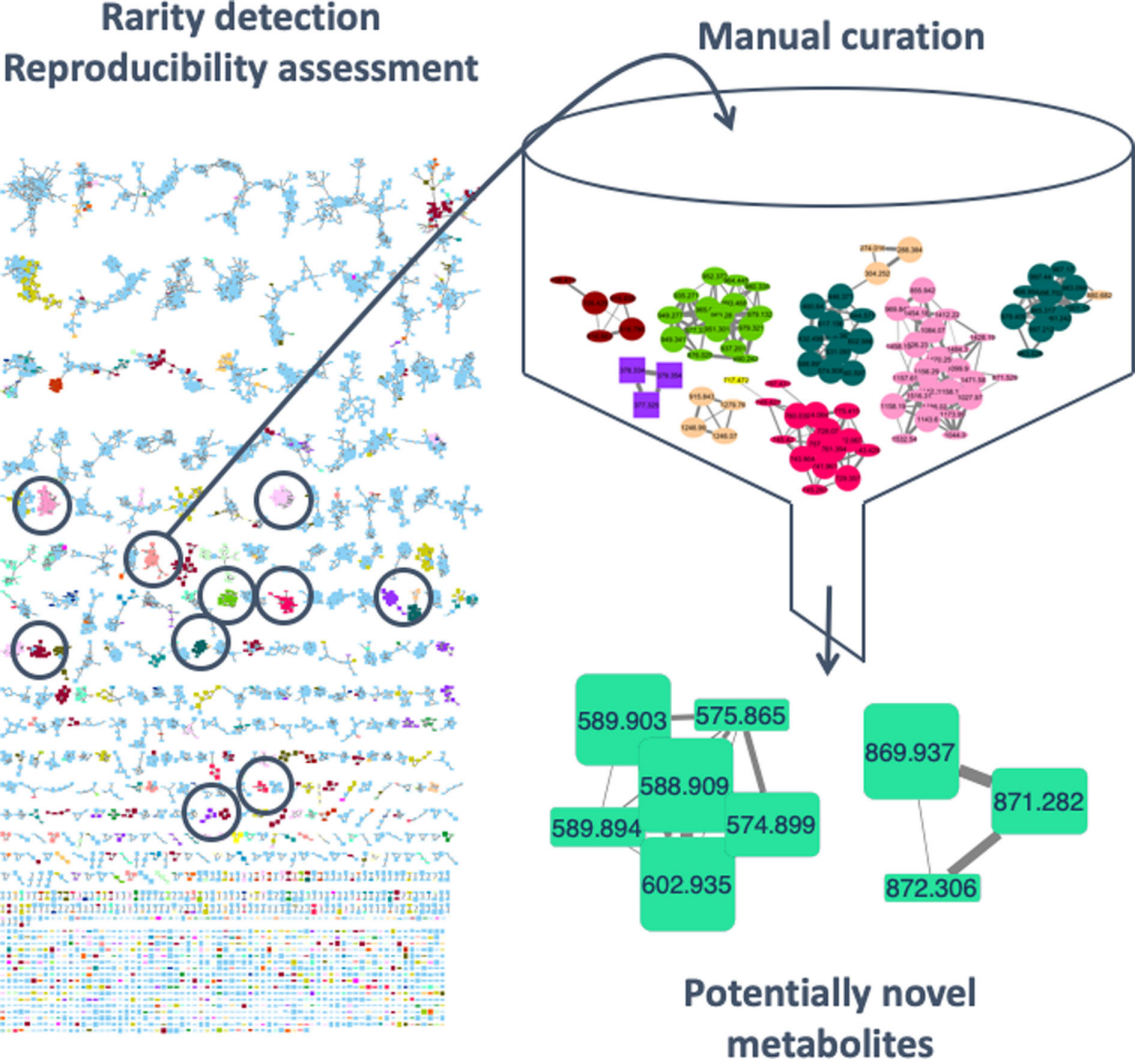
Rarity-based approach for finding novel metabolites. Families identified by molecular networking were searched for rarity and reproducibility (left portion), followed by manual curation, leading to the identification of two molecular families (bottom right).

The approach we followed is illustrated in Fig. 3 and summarized below. Essentially, we prioritized signals on the basis of two criteria: rarity and reproducibility. Rarity was assessed by a multistep search, in which we compared the 21-strain dataset against the 14 000-extract library through GNPS (Wang et al., 2016) and MZmine (Pluskal et al., 2010). We first started from a GNPS-generated network to look for *m/z* values and MS/MS fragmentation patterns absent in the extract library, then excluded molecular families derived from more than 10 strains. This led to 27 potentially interesting metabolites belonging to 21 different molecular families. These signals were then manually curated, selecting those that appeared in noncrowded regions of the chromatogram and were associated with a detectable UV–Vis spectrum. Then, MZmine was used to look for similar *m/z* values and retention times, thus considering those metabolites that, lacking MS/MS fragmentation, are missed by GNPS. After inspection of the raw data for consistency, we included a reproducibility criterion, which required that the potentially interesting metabolites were present in extracts prepared from at least three independent cultivations of the same strain (i.e., biological replicates), leading to two candidates (Fig. 3) selected for further characterization (K.V. et al., unpublished results).

This work highlighted the value of looking for infrequent but reproducible events to pinpoint potentially novel chemistry. Consistent with the prediction, both the selected metabolites did turn out to be novel and one did possess antibacterial activity under appropriate conditions (K.V. et al., unpublished results). The rarity-based approach has been applied also to the *Planomonospora* dataset, as described below (Zdouc et al., 2021).

## A Look at a Talented Strain

When examining the molecular fingerprints obtained from the extracts, we noticed that a subset of strains produced complex fingerprints with several different, apparently unrelated compounds. One such “talented” strain is *Streptomyces* sp. ID38640, which produces pseudouridimycin (PUM), a nucleoside analog inhibitor of RNA polymerase (Maffioli et al., 2017). We previously reported that this strain produces the structurally unrelated metabolites desferroxiamine, a siderophore, and lydicamycin, a linear chain polyketide. Intriguingly, their levels were altered in different *pum* mutants (Sosio et al., 2018).

**Fig. 4:**
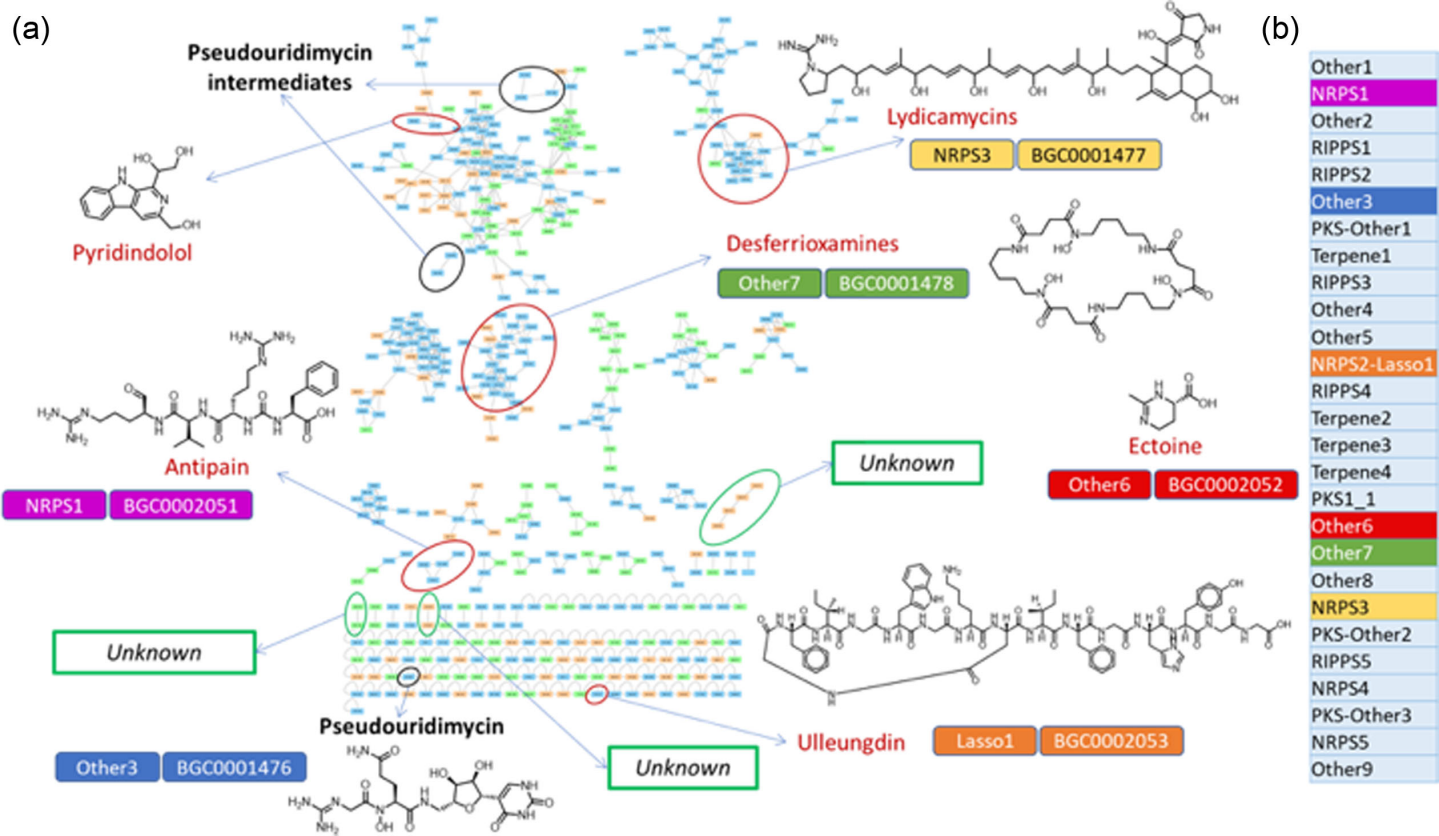
Metabolite investigation of the pseudouridimycin producer *Streptomyces* sp. ID38640. (a) Complete molecular network of 44 extracts from the wild-type strain and its knockout *pum* mutant strains. The network encompasses 475 features (= nodes), of which 369 were organized in 36 molecular families. Node colors give the contributing medium: orange for M8, green for PumP1, light blue for both. Black circles indicate PUM-related nodes, red circles indicate nodes corresponding to known compounds, green circles show potentially novel metabolites. The identified biosynthetic gene clusters are shown next to each metabolite, while the AntiSMASH (Montalbán-López et al., 2021) output of the entire genome in panel B. Note that ectoine is not present in the molecular network because of its limited number of MS/MS fragments. See Iorio et al. for further details (Iorio et al., 2021).

We recently expanded this work after cultivating several *pum* mutants and the wild type strain under different conditions (Iorio et al., 2021). Analysis by molecular networking ( Wang et al., 2016) of the corresponding extracts is shown in Fig. 4a. The analysis was performed using the wild type strain and 10 knockout mutants blocked in PUM biosynthesis (Sosio et al., 2018; Iorio et al., 2021). The strains were cultivated in two different media and analyzed at a single time point. Analysis of selected network families enabled identification of additional metabolites, which were then associated to the corresponding biosynthetic gene cluster (BGC) in the *Streptomyces* sp. ID38640 genome (Fig. 4a). The identified metabolites include: the ureylene-containing oligopeptide antipain, produced by nonribosomal peptide synthetases (NRPS) in numerous bacteria and acting as a protease inhibitor (Zdouc et al., 2021); the recently described lassopeptide ulleungdin (Son et al., 2018); ectoine, a tetrahydropyrimidinecarboxylic acid common in many bacterial species where it acts as an osmoprotectant; and pyridindolol, along with its glycosylated form, a β-carboline derivative produced by *Streptomyces* and reported as a β-galactosidase inhibitor (Aoyagi et al., 1975). We identified three additional, distinct signals (Fig. 4a, green boxes) that do not cluster to any signal present in the 14 000 extracts of Fig. 1b (and are thus rare) and could not be matched to any known metabolite (and are thus likely to be novel). Overall, our analyses allowed correlation between seven BGCs and the corresponding metabolites (Fig. 4b).

Some metabolites, which were not detected or produced in trace amounts only in the wild type strain, were clearly visible in one or more of the *pum* mutants (Iorio et al., 2021). This suggests an additional strategy for triggering expression of silent BGCs. In any case, *Streptomyces* sp. ID38640 appears as a prolific producer of several metabolites, with seven metabolite families identified and three more awaiting further investigations.

## A Look at a “Rare” Genus

As mentioned above, the NAICONS library contains strains belonging to many different actinomycete genera, most of which have not been systematically evaluated for metabolite richness and diversity. One such genus is *Planomonospora*, originally described in 1967 by microbiologists working at Lepetit (Thiemann et al., 1967). Since then, only a limited number of species have been formally described (Vobis et al., 2015) and just a handful of metabolites have been reported: the ribosomally synthesized, post-translationally modified thiopeptide (RiPPs) siomycin (Thiemann et al., 1968), the lanthipeptide 97518/planosporicin (Maffioli et al., 2009), the lassopeptide sphaericin (Kodani et al., 2017), and the NRPS-produced oligopeptide antipain (Wingender et al., 1975). Similarly, before our work, only one genome sequence was available (Dohra et al., 2016). Such limited information could derive from difficulties in isolating *Planomonospora* strains from environmental samples and/or from a limited metabolic versatility of this genus.

**Fig. 5:**
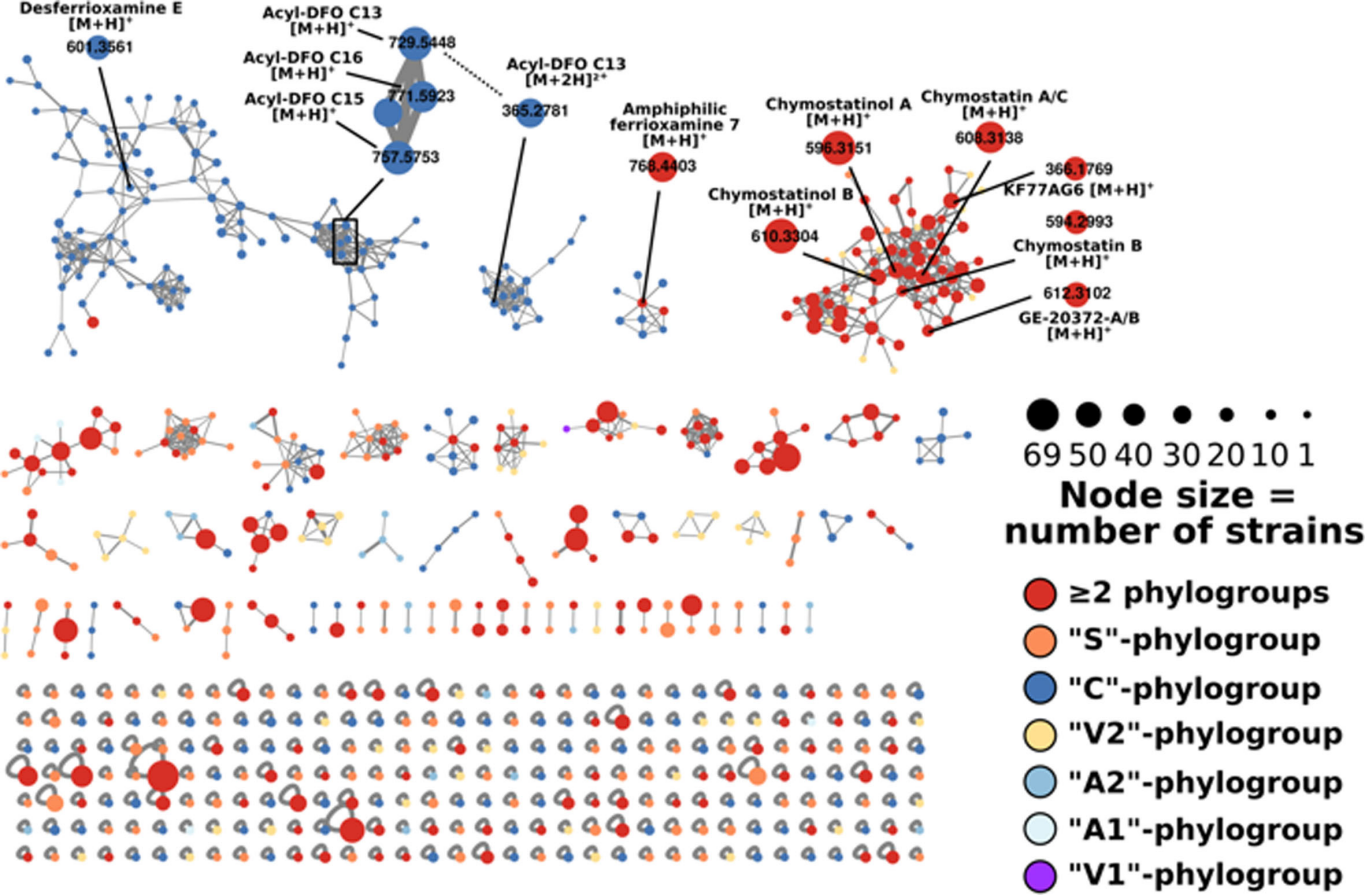
Complete molecular network of 286 *Planomonospora* extracts (obtained from 72 strains) and selected annotated molecular families. Node size correlates to the number of contributing strains, while the colors give the contributing phylogroup(s). Adapted from Zdouc et al. (2021).

We recently performed a metabolomic study of 72 *Planomonospora* isolates from NAICONS strain library, along with phylogenetic analysis of the corresponding 16S rRNA gene sequences and genomic analysis of selected strains (Zdouc et al., 2021). In the metabolomic dataset, we identified 60 distinct molecular families, in addition to a large number of singletons (Zdouc et al., 2021). We were able to confirm the presence of the three RiPP (siomycin, 97518, and sphaericin) and the single nonribosomal peptide (antipain) previously observed in *Planomonospora* strains. In addition, we found that members of this genus produce as main siderophore either desferrioxamine or compounds related to the nonribosomal tetrapeptides salinichelin and erythrochelin (Fig. 5). The presence of alternate siderophores in an actinobacterial genus has been previously observed (Bruns et al., 2018). Our data also indicated a strong correlation between chemical diversity and strain phylogeny, with the thiopeptide siomycin being observed in all strains belonging to just one phylogenetic branch but not in any other (Zdouc et al., 2021).

The number of BGCs identified in the three newly sequenced *Planomonospora* genomes ranged between 23 and 28, which could be grouped into 49 gene cluster families. The analysis pointed to the existence of a single BGC of unknown function that was common among all five *Planomonospora* genomes (Zdouc et al., 2021).

**Fig. 6:**
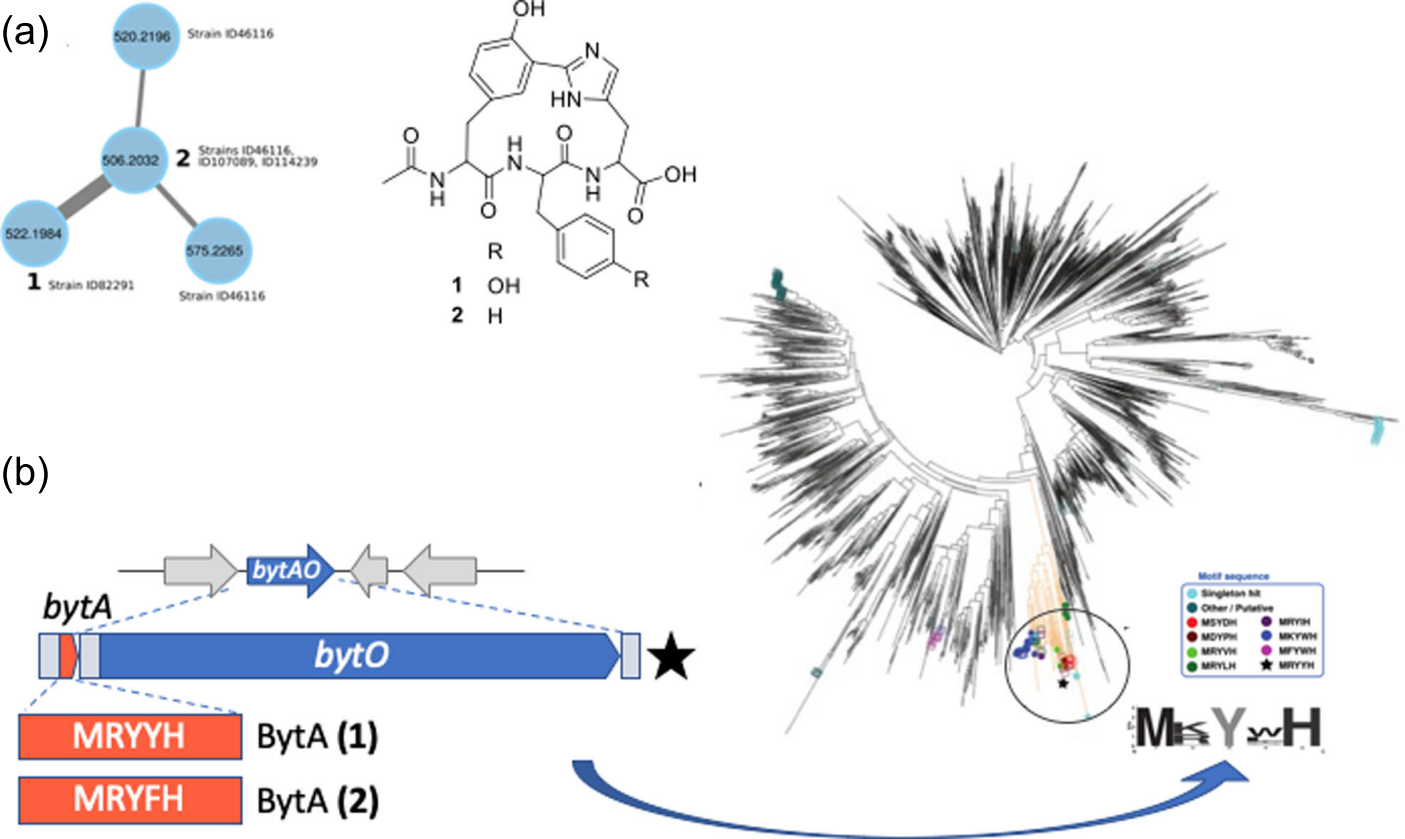
The discovery of biarylitides. (a) Molecular network analysis showing the molecular family consisting of biarylitides YYH (**1**; *m/z* 522.19) and YFH (**2**; *m/z* 506.2), and the corresponding chemical structures. (b) Left portion, the *bytAO*-containing region of the *Planomonospora* sp. ID82291 genome. *bytA* encodes the 5-aa precursor peptide of **1** and *bytO* encodes a cytochrome P450 monooxygenase. The 5-aa precursor peptide of **2** from the *Planomonospora* sp. ID107089 genome is shown below. The DNA segment marked by a start was used for heterologous expression (Zdouc et al., 2020). Right portion: phylogenomic analysis of BytO-related sequences, with the clade containing closely linked pentapeptide-encoding genes shown in orange and highlighted by the circle. The frequency of amino acid occurrence in the pentapeptides at the bottom right. See Zdouc et al. (2020) for further details.

Most of the molecular families identified in the *Planomonospora* metabolome could not be assigned to known compounds (Zdouc et al., 2021). Similarly, most of the BGCs present in the three analyzed *Planomonospora* genomes did not match BGCs specifying for known molecules (Zdouc et al., 2021). Following a rarity-based approach similar to the one described above, we were able to identify by molecular networking a group of three related signals observed in samples derived from four distinct but phylogenetically related strains (Fig. 6a). Following purification and structural elucidation, the corresponding molecules were established to represent *N*-acetylated tripeptides carrying an unprecedented Tyr-His biaryl cross link between amino acids 1 and 3, with the second amino acid being Tyr in one strain and Phe in the other three (Fig. 6a). We have proposed the name biarylitides for these compounds (Zdouc et al., 2020).

From the genomes of two *Planomosospora* strains, the cognate BGCs were found to consist of just two genes: *bytO*, encoding a cytochrome P450 monooxygenase likely responsible for installing the cross-link, and the closely *bytA*, encoding a pentapeptide with the mature peptide at the C-terminus. *bytA* represents the shortest known gene (Fig. 6b). Heterologous expression in *Streptomyces coelicolor* confirmed the production of mature biarylitide-YYH by the minimal gene cluster *bytAO* (Zdouc et al., 2020). While this work established the ribosomal synthesis of biarylitides, it provides to our knowledge the first example of a RiPP lacking a canonical leader sequence of about 20 amino acids, which has been established to be the critical recognition element for many enzymes involved in maturation of the core peptide (Arnison et al., 2013; Montalbán-López et al., 2021).

Such a small BGC, with just one cytochrome P450 monooxygenase and a very short CDS missed by annotation algorithms, was not detected by antiSMASH (Blin et al., 2019), the workhorse of BGC analysis. Wondering whether similar BGCs existed in available sequence databases, we looked for homologs of BytO and closely linked pentapeptide-encoding sequences in microbial genomes. A search where the third and fifth amino acids were restricted to Tyr and Tyr/His, respectively, led to 200 occurrences, mostly in actinobacterial genomes (Fig. 6b). Pentapeptide-encoding genes preceded by a ribosomal binding site showed a strong preference for a basic amino acid at position 2 (Fig. 6b). Remarkably, some of the two-gene loci were closely linked to genes specifying enzymes that might participate in further processing of the peptide, such as methyltransferases, sulfotransferases, ATP-grasp enzymes, etc. (Zdouc et al., 2020). This suggests that the biarylitide family of peptides might provide additional variations on the tripeptide core.

## Conclusions and Future Perspectives

The selected examples reported above illustrate that metabolites remain to be discovered, even from extensively explored microbial sources such as streptomycetes. For those strains, looking for rare or unique MS signals seems promising, as it facilitates the identification of novel metabolites without placing historical biases. To this end, it greatly helps to have a large dataset for comparison, as represented by a 14 000-extract library (Fig. 1b). Other approaches illustrated here involve looking at metabolites after knocking down a biosynthetic route, which however requires genetic manipulation of the strain of interest, and looking at poorly investigated microbial taxa. In any case, searching for novel metabolites is akin to looking for the classical needle in a haystack, which requires the development of appropriate skills and a “feeling” for what is feasible to pursue.

## Data Availability

Access to selected data can be provided upon request.
